# Conditional promoters to investigate gene function during wheat infection by *Zymoseptoria tritici*

**DOI:** 10.1016/j.fgb.2020.103487

**Published:** 2021-01

**Authors:** Elena Fantozzi, Sreedhar Kilaru, Stuart Cannon, Martin Schuster, Sarah J. Gurr, Gero Steinberg

**Affiliations:** a School of Biosciences, University of Exeter, Exeter EX4 4QD, UK; b University of Utrecht, Padualaan 8, Utrecht 3584 CH, the Netherlands

**Keywords:** Septoria tritici leaf blotch, Plant-pathogen interaction, Conditional promoters, Plant cell wall-degrading enzymes

## Abstract

The fungus *Zymoseptoria tritici* causes Septoria tritici leaf blotch, which poses a serious threat to temperate-grown wheat. Recently, we described a raft of molecular tools to study the biology of this fungus *in vitro*. Amongst these are 5 conditional promoters (P*nar1*, P*ex1A*, P*icl1,* P*gal7,* P*laraB*), which allow controlled over-expression or repression of target genes in cells grown in liquid culture. However, their use in the host-pathogen interaction *in planta* was not tested. Here, we investigate the behaviour of these promoters by quantitative live cell imaging of green-fluorescent protein-expressing cells during 6 stages of the plant infection process. We show that P*nar1* and P*icl1* are repressed *in planta* and demonstrate their suitability for studying essential gene expression and function in plant colonisation. The promoters P*gal7* and P*ex1A* are not fully-repressed *in planta*, but are induced during pycnidiation. This indicates the presence of inducing galactose or xylose and/or arabinose, released from the plant cell wall by the activity of fungal hydrolases. In contrast, the P*laraB* promoter, which normally controls expression of an α-l-arabinofuranosidase B, is strongly induced inside the leaf. This suggests that the fungus is exposed to L-arabinose in the mesophyll apoplast. Taken together, this study establishes 2 repressible promoters (P*nar1* and P*icl1*) and three inducible promoters (P*gal7*, P*ex1A,* P*laraB*) for molecular studies *in planta*. Moreover, we provide circumstantial evidence for plant cell wall degradation during the biotrophic phase of *Z. tritici* infection.

## Highlights

• We analyze the activity of 5 conditional promoters in *Z. tritici*-infected wheat leaves.
• The promoters P*nar1* and P*icl1* are repressed *in planta.*
• The promoter P*laraB* is induced in hyphae during all *in planta* infection stages.
• The promoters P*gal7* and P*ex1A* are not tight, but induced during late infection.
• Conditional promoters respond to plant cell wall-derived sugars in the apoplast.

## 1 Introduction

The ascomycete fungus *Zymoseptoria tritici* causes Septoria leaf blotch (STB), the most devastating disease of temperate-grown wheat (Fones et al., 2020). Despite mitigation, STB causes 5–10% yield loss in European wheat, which equates to 240 million Euros per year in the UK alone, with an additional ~ 160 million Euros spent on fungicide treatment (Fones and Gurr, 2015). *Z. tritici* is a dimorphic fungus which grows saprophytically as multi-cellular “budding” spores, but switches to hyphal growth upon initiation of its pathogenic phase. The invasive fungal hyphae explore the leaf surface and enter the plant through stomata. Once inside the host, the fungal hyphae colonise the apoplastic space of the mesophyll. At the end of this 2–10 day biotrophic phase, fungal hyphae begin to line virgin stomatal cavities. This marks the beginning of the formation of asexual fruiting bodies, named pycnidia. This developmental step coincides with the initiation of a necrotrophic phase, characterised by the disintegration of plant tissues. The release of plant-derived nutrients is thought to fuel massive fungal proliferation, finally resulting in the release of large numbers of multi-cellular pycnidiospores from the fruiting body (for references and further information see Duncan and Howard, 2000, Kema et al., 1996, Rudd et al., 2015, Fantozzi et al., 2020).

Despite our growing knowledge of the host/pathogen interaction, our understanding of the cell biology of the pathogen *per se* and its infection strategy is in its infancy (Steinberg, 2015). To address this, we established molecular tools to study *Z. tritici* at the cellular level. These include red- and green-fluorescent proteins (Kilaru et al., 2015d, Schuster et al., 2015a) which, when fused to organelle-specific markers, allow live cell imaging of cellular responses to gene deletions; this provides functional insight (Guo et al., 2015, Kilaru et al., 2015c, Kilaru et al., 2017, Schuster et al., 2015b). However, this strategy is not applicable to essential genes, as deletion of these is lethal. This technical challenge can be overcome by the use of conditional promoters. Upon addition of expression-controlling molecules, such as sugars or nutrients, such promoters can be repressed, causing protein depletion, or induced, resulting in protein over-expression. Such promoters have been established in several plant pathogens (Bottin et al., 1996, Wang et al., 2003, Lee et al., 2010). Over-expression of a given protein can be used to understand protein function in the cell (Cairns et al., 2015, Van Zyl et al., 2014) or alter protein activity to reveal counter-acting molecules (Wedlich-Söldner et al., 2002). Moreover, protein–protein interactions can be interrupted by controlled overexpression of peptides, which again provides an alternative way to investigate the cellular role of a given protein (Schuster et al., 2011).

We recently reported the use of 5 repressible promoters in *Z. tritici* (Kilaru et al., 2015a); P*nar1*: controls expression of nitrate reductase (JGI identity number: 111003), P*laraB*: controls expression of α-l-arabinofuranosidase B (JGI ID: 70396), P*ex1A*: controls expression of 1,4-β-endoxylanase A (JGI ID: 60105); P*gal7*: controls expression of galactose-1-phosphate uridylyltransferase (JGI ID: 72281); P*icl1*: controls expression of isocitrate lyase (JGI ID: 102083). All of these promoters are repressed in liquid media, supplemented with glucose (P*gal7*, P*laraB*, P*icl1*), ammonium (P*nar1*), maltodextrin (P*ex1A*) or induced when cells are grown in the presence of galactose (P*gal7*), arabinose (P*laraB*), sodium acetate (P*icl1*), nitrogen (P*nar1*) or xylose (P*ex1A*). While these molecular tools are useful for the investigation of protein function *in vitro*, experimental control of these promoters during the host-pathogen interaction is not possible.

*Z. tritici* hyphae colonise the mesophyll of wheat leaves (Kema et al., 1996). Here, the fungus stays in the extra-cellular part of this parenchyma tissue, which includes the plant cell walls and the airspace between the adjacent mesophyll cells (Sattelmacher, 2001). In this apoplastic environment, the invading hyphae will encounter a cocktail of plant-derived molecules (Roman-Reyna and Rathjen, 2017, Wimmer et al., 2003), which could act as repressors or inducers of conditional promoters. Moreover, plant invasion and colonisation of the apoplast by *Z. tritici* is accompanied by its secretion of plant cell wall-degrading enzymes (PCWDEs; Brunner et al., 2013). These hydrolases digest plant cell wall polysaccharides, thereby releasing mono-saccharides into the apoplast, which could themselves act as inducers or repressors of conditional promoters. Thus, a given promoter may encounter inducing and repressing cues at the same time, which makes the outcome for controlled gene expression unpredictable.

In this study, we monitor cytoplasmic GFP expression in hyphae of *Z. tritici* under 5 conditional promoters during plant invasion and colonisation. We measure cytoplasmic fluorescent signal intensity in *Z. tritici* during 6 stages of the infection process (Fantozzi et al., 2020), beginning with the persistence of the spore on the leaf cuticular layer and ending with macropycnidia formation in asexual fruiting bodies. This reveals that P*nar1* and P*icl1* are suitable to repress target gene expression during plant colonisation and pycnidiation, whereas P*ex1A* and P*gal7* are only partially repressed or even induced in these infection-related stages. Expression of GFP under P*laraB* was strongly induced during the biotrophic phase and pycnidiation *in planta*, suggesting that plant cell wall degradation by the invading hyphae releases L-arabinose. Thus, P*nar1*, P*icl1* and P*laraB* are of particular use to investigate the molecular environment of the wheat-*Z. tritici* pathosystem.

## 2 Materials and methods

### 2.1 Predicted expression of PCWDEs during wheat infection

We determined the transcription profile of classes of putative plant cell wall degrading enzymes (n = 49; Brunner et al., 2013, Rudd et al., 2015, Yang et al., 2013) over the *Z. tritici* infection cycle. To this end, we extracted the normalised expression (FPKM/RPKM) values from published data sets for each gene from RNASeq experiments using infected wheat leaves at days 1, 4, 9, 14, 21 (Rudd et al., 2015), 7 and 13 (Brunner et al., 2013) post infection. Initially, we investigated the peak expression of each enzyme within the time points 1–4, 5–10 and > 10 days post inoculation (Table 1). For each enzyme, the time point with the highest mean FPKM/RPKM expression was set to 100% and the two other time points were calculated relative to this value (Table 1).

**Table 1:** PCWDEs shown experimentally to be involved in wheat leaf infection.

### 2.2 Bacterial and fungal strains and growth conditions

*Escherichia coli* strain DH5α was used for the propagation of plasmids. *A. tumefaciens* strain EHA105 (Hood et al., 1993) was used for *A. tumefaciens*-mediated transformation of *Z. tritici*. *E. coli* and *A. tumefaciens* were grown in DYT media (Kilaru et al., 2015b) at 37 °C and 28 °C respectively. The fully sequenced *Z. tritici* wild-type isolate IPO323 (from Centraalbureau voor Schimmelcultures, Utrecht, The Netherlands; CBS 115943; Goodwin et al., 2011, Kema and van Silfhout, 1997) served as recipient strain for the genetic transformation experiments. *Z. tritici* strains were inoculated from stocks stored in NSY glycerol at −80 °C onto solid YPD agar (Kilaru et al., 2015b) and grown at 18 °C for 4–5d. The genotype of all *Z. tritici* strains is listed in Table 2. The experimental usage of all strains is summarised in Supplementary Table 1.

**Table 2:** Strains and plasmids used in this study.

**Strain name**	**Genotype**	**Reference**
IPO323_P*nar1*G_mChSso1	*MAT1-1*/pCP*nar1*eGFP/pHmCherrySso1, *cbx ^R^, hyg^R^*	This study
IPO323_P*icl1*G_mChSso1	*MAT1-1*/pCP*icl1*eGFP/pHmCherrySso1, *cbx ^R^, hyg^R^*	This study
IPO323_G	*MAT1-1*/pCeGFP, *cbx ^R^*	(Kilaru et al., 2015b)
IPO323_ZtG	*MAT1-1*/pCZtGFP, *cbx ^R^*	(Kilaru et al., 2015d)
IPO323_ZtG_P*nar1*Tub2	*MAT1-1*/pCZtGFP/P*nar1-tub2, cbx ^R^, hyg^R^*	This study
IPO323_P*gal7*G_mChSso1	*MAT1-1*/pCP*gal7*eGFP/pHmCherrySso1, *cbx ^R^, hyg^R^*	This study
IPO323_P*ex1A*G_mChSso1	*MAT1-1*/pCP*ex1A*eGFP/pGmCherrySso1, *cbx ^R^, G418^R^*	This study
IPO323_P*laraB*G_mChSso1	*MAT1-1*/ pCP*laraB*eGFP/pHmCherrySso1, *cbx ^R^, hyg^R^*	This study
pCP*nar1*eGFP	*Pnar1-egfp, cbx^R^*	(Kilaru et al., 2015a)
pHmCherrySso1	*Ptub2-mCherry-sso1, hyg^R^*	(Kilaru et al., 2017)
pCP*icl1*eGFP	*Picl1-egfp, cbx^R^*	(Kilaru et al., 2015a)
pCeGFP	*Ptub2-egfp, cbx^R^*	(Kilaru et al., 2015b)
pCZtGFP	*Ptub2-ztgfp, cbx^R^*	(Kilaru et al., 2015d)
pHP*nar1*Tub2	*Pnar1-tub2, hyg^R^*	This study
pCP*gal7*eGFP	*Pgal7-egfp, cbx^R^*	(Kilaru et al., 2015a)
pCP*ex1A*eGFP	*Pex1A-egfp, cbx^R^*	(Kilaru et al., 2015a)
pGmCherrySso1	*Ptub2-mCherry-sso1, G418^R^*	(Kilaru et al., 2017)
pCP*laraB*eGFP	*PlaraB-egfp, cbx^R^*	(Kilaru et al., 2015a)

MAT: mating type; -: fusion; /: ectopically integrated; P: promoter; -G or eGFP: enhanced green fluorescent protein; mCh or mCherry: monomeric red-fluorescent cherry protein; p: plasmid; *nar1*: nitrate reductase; Sso1: a syntaxin-like plasma membrane protein; C *or cbx^R^*: carboxin resistance; H or *hyg^R^*: hygromycin resistance; *icl1*: isocitrate lyase; ZtG or ZtGFP: codon-optimised green-fluorescent protein; *tub2*: α-tubulin; *gal7*: galactose-1-phosphate uridylyltransferase 7; *ex1A*: 1,4-b-endoxylanase; G or *G418^R^*: geneticin resistance; *laraB*: L-arabinofuranosidase B.

Growth conditions for induction and repression of promoters in *Z. tritici* strains were as follows: (a) IPO323_P*nar1*G_mChSso1 was grown in nitrate minimal medium (NM: Kilaru et al., 2015a), supplemented with 1% (w v^−1^) glucose (“ON” condition). To repress P*nar1* the strain IPO323_P*nar1*G_mChSso1 was grown in Complete medium (CM; Kilaru et al., 2015a; Holliday, 1974) with 1% (w v^−1^) glucose (“OFF” condition). (b) Expression of P*icl1* in cells of strain IPO323_P*icl1*G_mChSso1 was induced by growth in minimal medium (MM: Kilaru et al., 2015a), supplemented with 20 mM sodium acetate (“ON” condition) or with 2% (w v^−1^) glucose (“OFF” condition). (c) IPO323_P*gal7*G_mChSso1 cells were either grown in MM, supplemented with 0.5% (w v^−1^) galactose (“ON” condition) or with 2% (w v^−1^) glucose (“OFF” condition). (d) Cells of strain IPO323_P*ex1A*G_mChSso1 were grown in MM either supplemented with 5% (w v^−1^) xylose (“ON” condition) or 5% (w v^−1^) maltodextrin (“OFF” condition). (e) IPO323_P*laraB*G_mChSso1 cells were grown in MM, supplemented with 2% (w v^−1^) arabinose (“ON” condition) or 1% (w v^−1^) glucose (“OFF” condition). (f) IPO323_ZtG_P*nar1*Tub2 cells were grown in YPD liquid medium supplemented with 30 mM KNO_3_. All cultures were grown in 15 ml baffled flasks (DURAN, Wertheim, Germany) for 2 days at 200 rpm and 18 °C.

### 2.3 Molecular cloning

Vector pHP*nar1*Tub2 contains the *Z. tritici nar1* promoter, fused to the *tub2* gene. It is designed to replace the endogenous *tub2* by the *nar1* promoter, using hygromycin as the selection agent. This vector was generated by *in vivo* recombination in *S. cerevisiae* DS94 (MATα, *ura3-52*, *trp1-1*, *leu2-3*, *his3-111* and *lys2-801*) following published procedures (Kilaru and Steinberg, 2015). A 9533 bp fragment of pCeGFPTub2 (Schuster et al., 2015a; digested with *Bam*HI and *Hind*III), 1149 bp *tub2* promoter (amplified with primers SK-Sep-217 and SK-Sep-218 from vector pCeGFPTub2; see Supplementary Table 2 for all cloning primers),1806 bp hygromycin resistance cassette (amplified with primers SK-Sep-136 and SK-Sep-137 from vector pCHyg), 1000 bp *nar1* promoter (amplified with primers SK-Sep-219 and SK-Sep-220 from IPO323 genomic DNA), 5′ end of 1002 bp *tub2* gene (amplified with primers SK-Sep-221 and SK-Sep-222 from IPO323 genomic DNA) were recombined in *S. cerevisiae* to obtain the vector pHP*nar1*Tub2. The vector was transformed into *A. tumefaciens* strain EHA105 by a heat shock method (Holsters et al., 1978).

### 2.4 *Z. tritici* transformation and molecular analysis of transformants

*A. tumefaciens* mediated transformation of *Z. tritici* was performed as described previously (Kilaru et al., 2015b). In order to visualise the plasma membrane in *Z. tritici* hyphae, either the vector pHmCherrySso1 or pGmCherrySso1 (Kilaru et al., 2017) containing *mCherry* fused to the full-length *sso1* gene under the control of constitutive *tub2* promoter, was randomly integrated into the genome of strains IPO323_P*nar1*G, IPO323_P*icl1*G, IPO323_P*gal7*G, IPO323_P*ex1A*G and IPO323_P*laraB*G (Kilaru et al., 2015a), using either hygromycin or G418 as selection agent. See Table 2 for the genotype of the resulting strains IPO323_P*nar1*G_mChSso1, IPO323_P*icl1*G_mChSso1, IPO323_P*gal7*G_mChSso1, IPO323_P*ex1A*G_mChSso1 and IPO323_P*laraB*G_mChSso1.

To generate a conditional α-tubulin (*tub2*) mutant in *Z. tritici*, vector pHP*nar1*Tub2 was integrated into the genome of strain IPO323_ZtG (Kilaru et al., 2015d), resulting in strain IPO323_ZtG_P*nar1*Tub2. The *nar1* promoter was induced by addition of 30 mM KNO_3_ (Sigma–Aldrich, Gillingham, UK) to the *Agrobacterium* induction medium (Kilaru et al., 2015b) supplemented with 200 µM acetosyringone (Sigma–Aldrich) agar plates and grown at 18 °C for 3d. For selection of transformants, membranes were transferred to Czapek dox agar plates (35 g l^−1^ Czapek Dox broth [Oxoid, Basingstoke, UK], 20 g l^−1^ agar, 30 mM KNO_3_). In addition, plates were supplemented with 200 µg/ml hygromycin (Roche, Burgess Hill, UK), 100 µg/ml cefotaxime and 100 µg/ml timentin (Melford, Ipswich, UK). After 12 day growth at 18 °C, individual transformants were transferred to YPD agar plates, supplemented with 30 mM KNO_3_ and incubated at 18 °C for additional 3–4d. Correct integration of the vector into the *tub2* locus was confirmed by Southern analysis.

### 2.5 Plate growth assay

YPD agar alone or YPD agar, supplemented with 30 mM KNO_3_, were used to examine growth of IPO323_ZtG and IPO323_ZtG_P*nar1*Tub2. Cells were grown in YPD/30 mM KNO_3_ at 18 °C, 200 rpm for 3d. Cell density was adjusted to 1x 10^6^ cells ml^−1^, using Cellometer Auto 1000 cell counter (Nexcelom Biosciences, Lawrence, USA). 5 µl cell suspension was placed on agar plates, followed by growth at 18 °C for 6d. Colony formation on YPD or YPD/30 mM KNO_3_ agar plates was documented using a Canon digital IXUS 80 IS camera (Canon, Surrey, UK).

### 2.6 Investigation of cytoplasmic eGFP expression in infected plant tissue

Cells were harvested from cultures, grown in “ON” or “OFF” conditions (see above), by centrifugation at 3000 rpm for 5 min. After washing twice with ddH_2_O, cell density was adjusted to 1 × 10^6^ cells ml^−1^ using Cellometer Auto 1000 cell counter (Nexcelom Biosciences) and cells were manually spread over wheat leaves using a glove (Fantozzi et al. 2020). The infected plants were transferred in a Fitotron SGC120 growth chamber (Weiss Technik UK, Loughborough, UK) under 14 h light (intensity 500 μmol of PAR), 24 °C, 80% RH (relative humidity); 10 h dark, 20 °C, 80% RH with automated watering. Plants were bagged with a transparent bag for the first 3 days to retain humidity. Plant tissue samples were collected every 2–3 days and prepared for microscopic analysis. Spinning disc microscopy used a motorised inverted microscope (IX81; Olympus, Hamburg, Germany), equipped with a UPlanSApo 60x/1.35 OIL objective (Olympus, Hamburg, Germany) and a CSU-X1 Spinning Disk head (Yokogawa), coupled with a CoolSNAP HQ2 CCD camera (Photometrics /Roper Scientific, Tucson, USA). Fluorescence was detected with an eGFP ET filter-set (470/40 ET Bandpass filter, Beamsplitter T 495 LPXR and 525/50 ET Bandpass filter (Chroma Technology GmbH, Olching, Germany) and a mCherry filter set. Illumination of fluorescent protein tags used a 488 nm laser (70 mW) or a 561 nm laser (70 mW). eGFP signals were acquired at various laser intensities (percent output power: P*nar* 1, 80%; P*ex1A*, 80%; P*laraB*, 30%; P*gal7*, 80%; P*icl1*, 30%) and 400 ms exposure time. mCherry signals were acquired at 100% output power and 600 ms exposure time. Images were analysed using the software package Metamorph 7.8x (Molecular Devices, Wokingham, UK) as described in Kilaru et al. (2015a).

### 2.7 Testing the effect of various sugars on promoter regulation

Strains IPO323_P*icl1*G_mChSso1, IPO323_P*gal7*G_mChSso1, IPO323_P*ex1A*G_mChSso1 and IPO323_P*laraB*G_mChSso1 were grown in minimal medium without carbon source or supplemented with 2% (w v^−1^) glucose, galactose, fructose, sucrose, xylose, L-rhamnose and L-arabinose (Sigma–Aldrich) for 2 days at 18 °C, 200 rpm. Aliquots of these cultures were investigated using laser-supported epifluorescence as described previously (Schuster et al., 2015a). 14-bit images were acquired at various laser intensities (percent output power: P*ex1A*, 30%; P*laraB*, 100%; P*gal7*, 40%; P*icl1*, 20%) and 300 ms exposure time. Signal intensities in these images were measured using the software package Metamorph 7.8x.

### 2.8 Plant infection assays

Wheat leaf infections were performed, as described previously (Steinberg et al., 2020), using 12-14 day-old plants (winter wheat *Triticum aestivum*, cultivar Galaxie). Cells of *Z. tritici* strains IPO323_ZtG and IPO323_ZtG_P*nar1*Tub2 were grown as described above. For infection, 1 ml of cell suspension was pre-adjusted to 1 × 10^6^ cells ml^−1^ using Cellometer Auto 1000 cell counter (Nexcelom Biosciences) and sprayed at 5psi using a Voilamart AS18 airbrush/compressor (Hydirect, Guang Dong, China) onto the second leaf. Plants were covered by transparent plastic bags for 3 days and incubated in a growth chamber as described above. After 18 days, second leaves were detached and incubated for 3 days in a sealed container in high humidity at 25 °C. Leaves were scanned using an Epson Perfection V850 Pro scanner (Epson, Hemel Hempstead, UK). The coverage of melanised pycnidia was determined using the software ImageJ (https://imagej.net/Downloads) and compared to total leaf area, following previous protocols (Steinberg et al., 2020).

## 3 Results and discussion

### 3.1 *Z. tritici* hydrolases are predicted to release mono-saccharides into the apoplast

**Fig. 1:**
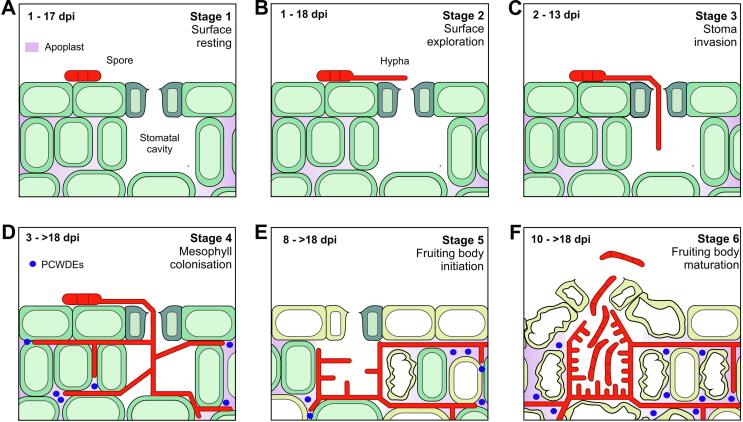
**Infection stages of *Z. tritici* in wheat.** (**A**) Stage 1, “Surface resting”. Spores can rest for an extended time on the leaf surface before switching to hyphal growth. (**B**) Stage 2, “Surface exploration”. After the morphogenic transition to hyphal growth, hyphae grow on the surface of the plant epidermis, thereby randomly “finding” stomata (Fones et al., 2017). (**C**) Stage 3, “Stoma invasion”. Hyphae enter the open stomatal aperture into the substomatal cavity. This penetration event was reported to occur for up to 13 dpi (Fantozzi et al., 2020). (**D**) Stage 4, “Mesophyll colonisation”. Once inside the plant tissue, hyphae branch and colonise the apoplastic space of the mesophyll, which includes the intercellular airspace and the plant cell walls. During this infection phase, the fungus secretes PCWDEs (blue dots; see Table 1). (**E**) Stage 5, “Fruiting body initiation”. When reaching new stomata, colonising hyphae begin to line the cavity and fill the space under the stoma with hyphae. The formation of fruiting bodies coincides with first signs of chlorosis (yellow cells). (**F**) Stage 6, “Fruiting body maturation”. Hyphae have filled the substomatal cavity and the fruiting body (pycnidium) forms multi-cellular spores. Live cell imaging has estimated that each pycnidium releases ~ 300 spores (Fones and Gurr, 2015). At this stage, plant cells are undergoing programmed cell death (Keon et al., 2007), which is thought to be initiated by necrosis factors (Kettles and Kanyuka, 2016). The figure was modified from (Fantozzi et al., 2020). (For interpretation of the references to colour in this figure legend, the reader is referred to the web version of this article.)

Our previous work established 5 conditional promoters, which are inducible or repressible *in vitro* by the addition of nutrients or mono-saccharides (Kilaru et al., 2015a). However, such an approach is not possible *in planta*. After entry through stomata, *Z. tritici* hyphae colonise the apoplastic space of the infected wheat leaves (Fig. 1). We identified 49 genes encoding putative plant PCWDEs in the genome of *Z. tritici* (Brunner et al., 2013, Yang et al., 2013, Rudd et al., 2015). An increasing body of evidence shows that these hydrolases are expressed during the infection process (Brunner et al., 2013, Yang et al., 2013, Rudd et al., 2015, Haueisen et al., 2019) and secreted into the wheat leaf apoplast (M'Barek et al., 2015, Yang et al., 2015). The digestive activity of fungal hydrolases was shown to release mono-saccharides, such as xylose or L-arabinose from plant cell walls (Pogorelko et al., 2011). Such molecules could act as inducers of conditional promoters (e.g. P*ex1A*, Kilaru et al., 2015a). We therefore took a closer look at the transcription profiles of putative PCWDEs, using published RNAseq data sets (Brunner et al., 2013, Rudd et al., 2015). Indeed, we found support for the notion that inducing sugars, such as arabinose (induces P*laraB*), galactose (induces P*gal7*) and xylose (induces P*ex1A*) are produced by the activity of secreted PCWDEs during plant infection (Table 1). While transcription of PCWDE genes is not proof of their secretion and hydrolytic activity, these results suggest that invading fungal hyphae encounter a cocktail of molecules in the apoplast, which themselves may impact on the control of conditional promoters.

The sugar-regulated promoters used in this study have been reported to be induced by galactose (P*gal7*), arabinose (P*laraB*), sodium acetate (P*icl1*), or xylose (P*ex1A*; Kilaru et al. 2015a). Thus, GFP expression in a particular reporter strain inside *planta* could indicate the presence of a specific type of saccharide. This notion, however, requires a high specificity of the inducible promoters for sugar molecules. In fungi, monosaccharides control a raft of promoters (Kluge et al., 2018) and involves, cellular uptake of the sugar or signalling from plasma membrane-associated sugar sensors (overview Holsbeeks et al., 2004). Moreover, a sucrose transporter was reported in *U. maydis* (Wahl et al., 2010) and sucrose induces gene expression in a methylotrophic yeast (Puseenam et al., 2018). Thus, we considered it possible that sugars, present in the plant apoplast (sucrose, glucose, fructose; Naseem et al., 2017, Roman-Reyna and Rathjen, 2017) could act as inducers of the inducible promoters in *Z. tritici*. Moreover, enzymatic digestion of the plant cell wall would release additional saccharides, which includes galactose, arabinose, rhamnose and xylose (Scheller and Ulvskov, 2010, Jiang et al., 2020), which may also affect promoter activity.

**Fig. 2:**
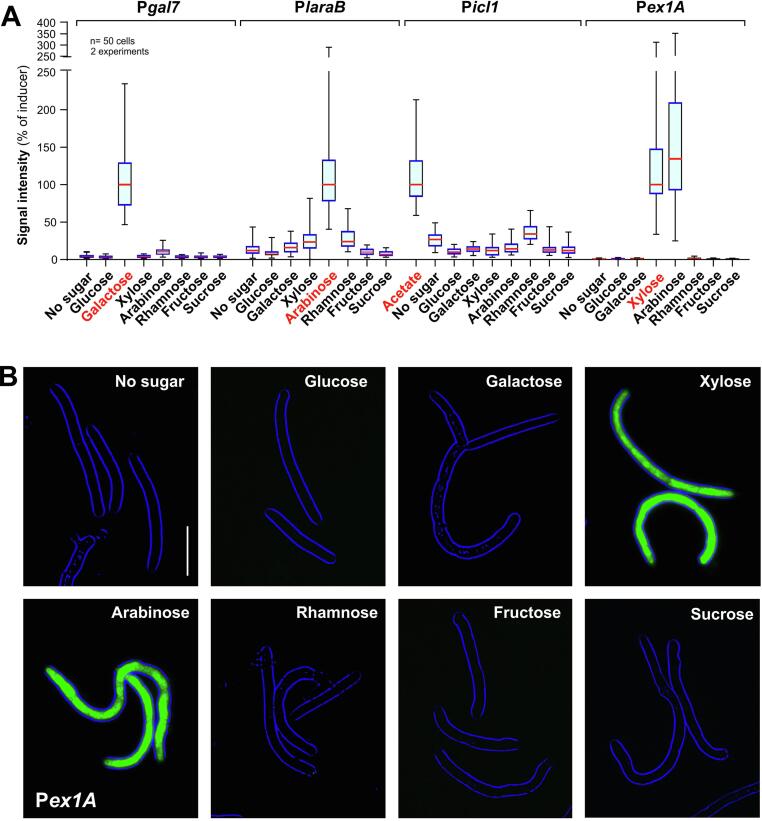
**Regulation of conditional promoters by various saccharides.** (**A**) Signal intensity of cytoplasmic eGFP, expressed under the conditional promoters P*gal7*, P*laraB*, P*icl1* and P*ex1A* after 2 days growth in minimal media, supplemented with 2% (w v^−1^) glucose, galactose, xylose, L-arabinose, L-rhamnose, fructose and sucrose. Several data sets are non-normally distributed (Shapiro -Wilk test, P > 0.05) and are therefore given as Whiskers' plots (blue lines: 25/75 percentiles; red line: median, whiskers: minimum and maximum values). Note that P*gal7* and P*icl1* are not tightly repressed in the presence of most sugars and that P*exA1* is induced by xylose and arabinose. Known promoter inducers are indicated in red. (**B**) Images showing induction of P*ex1A*, indicated by cytoplasmic eGFP expression, in *Z. tritici* cells, grown in minimal medium, supplemented with 2% xylose and 2% L-arabinose (w v^−1^). Cytoplasmic GFP fluorescence is shown in green, the edge of the spores is shown in blue. Scale bar represents 15 µm. (For interpretation of the references to colour in this figure legend, the reader is referred to the web version of this article.)

To test the response of the conditional promoters to these sugars, we grew our reporter strains in media, supplemented with 2% of each sugar and analysed GFP expression, detected by fluorescent microscopy. Consistent with our previous report (Kilaru et al., 2015a), we found that P*gal7* and P*laraB* are induced only by galactose and arabinose, respectively (Fig. 2a; P*gal7*, P*laraB*) and that P*icl1* was induced by acetate (Fig. 2a; P*icl1*). However, P*laraB* and P*icl1* showed low “background” expression of GFP, even in the presence of all sugars or even in the absence of any saccharide (Fig. 2a). In contrast, *Pex1A* did not express GFP expression under most conditions. Moreover, we found that *Pex1A* is not only induced by xylose, but also responds strongly to the presence of arabinose (Fig. 2a, 2b; *Pex1A*). Thus, the promoter of 1,4-β-endoxylanase A has at least two saccharide inducers, suggesting that GFP fluorescence in hyphae inside *planta* indicates the presence of xylose, arabinose or both.

### 3.2 The promoters P*nar1* and P*icl1* are repressed during the early infection

We next set out to investigate the expression activity of conditional promoters P*nar1*, P*icl1*, P*gal7*, P*ex1A* and P*laraB* during pathogenic development *in planta*. Recently, we used live cell imaging of *Z. tritici* to define 6 developmental stages during the infection process of *Z. tritici* in wheat leaves (Fantozzi et al., 2020; Fig. 1). To be able to locate fungal cells at each stage even in the absence of cytoplasmic GFP expression (e.g. when a given promoter is not active), we introduced a constitutively expressed red-fluorescent plasma membrane marker randomly into the genome of all reporter strains (see Table 2 for genotype of all strains). These strains continuously fluoresce red at the plasma membrane, but only show green-fluorescence in the cytoplasm upon induction of GFP-expression, driven by the conditional promoters.

We infected wheat leaves with these *Z. tritici* reporter strains. We took particular care to remove all traces of the growth medium by extensive washes of the spores before inoculating 12 day-old wheat leaves (see Methods). Subsequently, we acquired confocal images of cells every 2–3 days for up to 21 days, using spinning-disc confocal microscopy. Firstly, we located fungal cells by visualising mCherry-Sso1 red-fluorescence. Subsequently, we acquired the corresponding green-fluorescent images at defined laser settings and measured the fluorescent signal intensity in the cytoplasm in these images. It is important to note that we acquired images at all infection stages, visible at a given time-point, and combined them in the different stage categories.

**Fig. 3:**
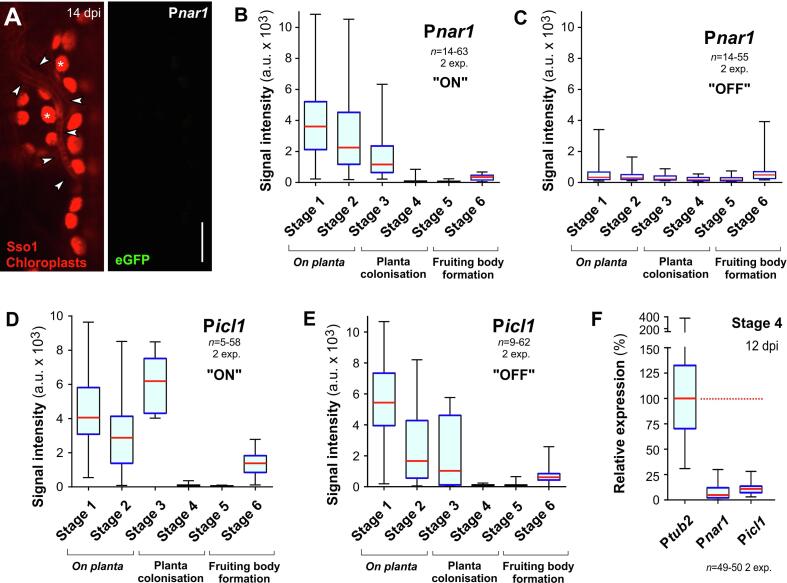
**The use of conditional promoters to repress gene expression *in planta*.** (**A**) Confocal images showing a hypha of strain IPO323_P*nar1*G_mChSso1 in wheat leaf tissue at 14dpi. The fungal plasma membrane is labelled with the red-fluorescent syntaxin mCherry-Sso1 (Kilaru et al., 2017; arrowheads in left image) and were co-detected with the auto-fluorescence of plant chloroplasts (asterisks in left panel). Due to repression of P*nar1*, cells do not express cytoplasmic eGFP (right panel, green). Scale bar represents 10 µm. (**B**) Whiskers' plot showing average signal intensities of cytoplasmic eGFP, expressed under P*nar1* that normally controls the expression of nitrate reductase, in the six stages of wheat leaf infection. Note that plants were infected with cells that were grown in “ON” condition, where NO_3_^–^ was provided as a nitrogen source. Sample size 14–63 structures from 2 experiments. (**C**) Whiskers' plot showing average signal intensities of cytoplasmic eGFP, expressed under P*nar1*, in the six stages of wheat leaf infection. Note that plants were infected with cells that were grown in “OFF” condition, where NH4^+^ was present in the medium. Note that the initial induction of cells on the plant surface is abolished. Sample size 14–55 structures from 2 experiments. (**D**) Whiskers' plot showing average signal intensities of cytoplasmic eGFP, expressed under P*icl1* that normally controls the expression of isocitrate lyase, in the six stages of wheat leaf infection. Note that plants were infected with cells that were grown in “ON” condition, where sodium acetate was provided as inducer. Sample size 5–58 structures from 2 experiments. (**E**) Whiskers' plot showing average signal intensities of cytoplasmic eGFP, expressed under P*icl1*, in the six stages of wheat leaf infection. Note that plants were infected with cells that were grown in “OFF” condition, where glucose was provided as carbon source. Note that the promoter was still induced in stage 1, suggesting the presence of acetate on the plant surface. Sample size 9–62 structures from 2 experiments. (**F**) Whiskers' plot showing relative signal intensities of cytoplasmic eGFP, expressed under P*nar1 and* P*icl1* relative to eGFP fluorescence, driven from the α-tubulin promoter P*tub2* (set to 100%, red dotted line). Cells were analysed in wheat leaf tissue at stage 4 (Mesophyll colonisation). All plant sample preparations, spinning-disc microscopy and measurements were done under identical condition to allow direct comparison between all strains. Sample size 49–50 structures from 2 experiments. As numerous data sets are non-normally distributed (Shapiro -Wilk test, P > 0.05), all data in (B-F) and are given as Whiskers' plots (blue lines: 25/75 percentiles; red line: median, whiskers: minimum and maximum values).

In a first set of experiments, we grew strain IPO323_P*nar1*G_mChSso1 for 48 h in nitrate minimal medium (MM), supplemented with 30 mM of the inducer molecule nitrate (NO_3_^–^; “ON” condition). Our previous work showed that P*nar1* was activated under these conditions, but repressed when grown in complete medium (CM; “OFF” condition; Kilaru et al., 2015a). The nitrate reductase promoter in various organisms was shown to be repressed by ammonium (NH_4_^+^, see (Li et al., 2007). CM contains 0.15% (w v^−1^) NH_4_^+^, which is most likely responsible for the repression of P*nar1* in this medium. Studies in tomato and oilseed rape leaves confirmed the presence of NH_4_^+^ in apoplastic fluid (Schjoerring et al., 2002), suggesting that P*nar1* in *Z. tritici* could be turned off after entering the wheat leaves tissue. Indeed, we found that GFP fluorescence decreases upon infection of leaves (Fig. 3A, 3B, Stage 3) and its expression is tightly repressed during colonisation and early fruiting body formation (Fig. 3B, Stage 4, 5). This result suggests that the fungal hyphae are exposed to apoplastic NH_4_^+^ in the mesophyll of wheat leaves. We also noticed strong expression of GFP on the plant surface (Stage 1 and Stage 2, Fig. 3B). We tested if this activation reflects access to NO_3_^–^ on the leaf surface, as shown in barley leaves (Nagai et al., 2013), or if it is due the inital “ON” condition during the culturing of the spores *in vitro*. We grew cells under “OFF” condition, where the promoter is repressed (Kilaru et al., 2015a), infected wheat leaves and measured GFP expression at the various infection stages. These experiments showed that P*nar1* was no longer induced in Stage 1 and Stage 2 (Fig. 3C). Thus, we consider it likely that the high activity of P*nar1* on plant surface is due to a “memory effect”, triggered by the presence of the inducer NO_3_^–^ in the inital liquid cultures.

Next, we tested the activity of the promoter for isocitrate lyase (P*icl1*) during wheat leaf infection. We reported that, under *in vitro* conditions, P*icl1* is induced by acetate (“ON”) and repressed by glucose (“OFF”; Kilaru et al., 2015a). IPO323_P*icl1*G_mChSso1 cells that were grown in “ON” condition, showed strong GFP expression in Stage 1–3, which dropped sharply when the hyphae colonised the mesophyll and began fruiting body formation (Fig. 3D; Stage 4, 5). The apoplastic fluid of wheat leaves contains glucose (Roman-Reyna & Rathjen, 2017). Moreover, the activity of secreted fungal cellulases, expressed during early stages of infection, likely increases glucose concentrations in the apoplast (Table 1). Thus, high glucose levels may repress P*icl1 in planta*. A similar result was reported for the promoter of isocitrate lysase (acu-3) from *Neurospora crassa*, which was also used heterologously in *Z. tritici* to detect GFP expression during infection (Rohel et al., 2001). We next asked if P*icl1* induction on the wheat leaf surface is due to a growth “medium memory effect”, as shown above for P*nar1*. When plants were infected with cells that were grown in “OFF” condition, P*icl1* was still strongly induced in Stage 1 under these conditions (Fig. 3E). This suggests that the fungus encounters acetate on the plant surface. Indeed, an extensive transcriptome study showed induction of key enzymes of the glyoxylate cycle at 1dpi and 4dpi (Rudd et al., 2015). This led to the conclusion that *Z. tritici* uses acetate, derived from metabolising cutin on the plant surface, for anabolic processes during the early phase of infection (Rudd et al., 2015). However, the sharp decline of P*icl1*-induced expression of eGFP in mesophyll-colonising hyphae (Fig. 3D, 3E, Stage 4) indicates that lipid metabolism via the glyoxylate cycle is not prominent during plant colonisation.

### 3.3 P*nar1*-controlled α-tubulin illustrates the use of conditional promoters in investigating essential genes during plant colonisation

**Fig. 4:**
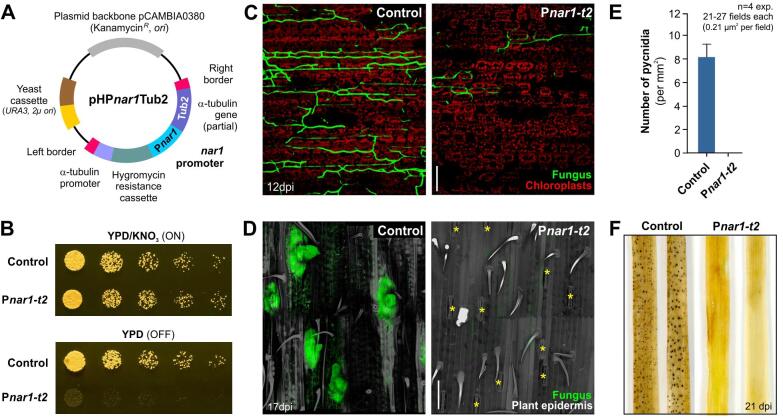
**The use of the promoter P*nar1* to study the role of the essential gene** α**-tubulin during *Z. tritici* plant colonization.** (**A**) Organisation of vector pHP*nar1*Tub2. The vector is designed for homologous integration of the inducible/repressible promoter P*nar1* in front of the open reading frame of the *Z. tritici* α-tubulin (*tub2*). Note that fragments are not drawn to scale. (**B**) Colony formation of conditional *Z. tritici* mutants, expressing the endogenous α-tubulin gene (*tub2*, Kilaru et al., 2015b) under the control of P*nar1.* Cells grow in the presence of NO_3_^–^ (YPD/KNO_3_; ON), when the essential *tub2* gene is expressed, but do not form colonies in the absence of this nitrogen source (YPD, OFF). Note that the presence of NH4^+^ actively represses P*nar1*. (**C**) Confocal images showing wheat leaf colonisation by strain IPO323_ZtG (Control) and strain IPO323_ZtG_P*nar1*Tub2, which expresses α-tubulin under the P*nar1* promoter (P*nar1-t2*) at 12dpi. Chloroplasts are detected by their auto-fluorescence (red) the fungus is detected by GFP-fluorescence (green). Scale bar represents 50 µm. (**D**) Confocal images of colonisation of the substomatal cavities in wheat leaves, infected with IPO323_ZtG (Control) and strain IPO323_ZtG_P*nar1*Tub2 (P*nar1-t2*) at 17 dpi. GFP fluorescence in the fungal cytoplasm is shown in green, the plant surface, detected by its auto-fluorescence, is shown in grey. Stomata in the right panel are highlighted by yellow asterisks. Scale bar represents 100 µm. (**E**) Percentage of pycnidia in wheat leaves, infected with IPO323_ZtG (Control) and strain IPO323_ZtG_P*nar1*Tub2 (P*nar1-t2*) at 21dpi. No pycnidia were found leaves infected with the conditional mutant. Bars represent mean ± SEM, sample size *n* = 4 experiments. (**F**) Septoria tritici blotch disease symptoms on wheat leaves at 21 days after infection with IPO323_ZtG (Control) and strain IPO323_ZtG_P*nar1*Tub2 (P*nar1-t2*). Note that the dark spots in Control leaves represent fungal fruiting bodies (pycnidia). Note that the yellow colour of leaves infected with strain IPO323_ZtG_P*nar1*Tub2 (P*nar-t2*) is most likely a consequence of the initial invasion of mutant hyphae, as such discolouration is only rarely found in negative control experiments, where leaves are treated only with 0.04% (v v^−1^) Tween 20, which breaks the surface tension during application of fungal spore suspensions onto wheat leaves. (For interpretation of the references to colour in this figure legend, the reader is referred to the web version of this article.)

Our results demonstrate that the promoters P*nar1* and P*icl1* are repressed in hyphae during the biotrophic phase (Stage 4 and 5). This suggests that these promoters could be used to investigate the role of essential genes *in planta*. To test this, we chose to analyse the role of α-tubulin (Tub2; Kilaru et al., 2015b) during plant colonisation. α-Tubulin is a crucial building block of microtubules (Desai and Mitchison, 1997), which are essential for fungal mitosis, hyphal growth and pathogenicity (Doshi et al., 1991, Fuchs et al., 2005, Horio and Oakley, 2005; Zhang et al., 2015). Such crucial functions underpin the fungicidal activity of tubulin-targeting benzimidazole fungicides (Davidse, 1986). To investigate the role of microtubules during plant colonisation in the *Z. tritici* pathosystem, we integrated a P*nar1*-carrying vector (Fig. 4A) into the endogenous *tub2* locus of strain IPO323_ZtG (Kilaru et al., 2015d). The resulting strain IPO323_ZtG_P*nar1*Tub2 showed normal growth on YPD agar plates, supplemented with 30 mM KNO_3_^−^ (“ON” condition), but it did not grow on YPD alone (Fig. 4B). This is most likely due to the repression of *tub2* expression by the presence of NH_4_^+^ in the media (0.08% (w v^−1^). Next, we infected wheat leaves with fungal cells grown under “ON” condition. We found that GFP-expressing hyphae enter the plant, but colonisation of the mesophyll is clearly reduced (Fig. 4C; Stage 3). No signs of later stages of infection, such as stomatal cavity lining (stage 5) and fruiting body formation (stage 6; Fig. 4D) were found in these experiments (Fig. 4D, 4E). As a consequence, no mature black pycnidia are formed on IPO323_ZtG_P*nar1*Tub2-infected wheat leaves (Fig. 4F). However, all leaves, infected with the mutant, showed yellow chlorotic tissue (Fig. 4F, Supplementary Fig. 1). As such, chlorosis was only rarely observed when leaves were treated with 0.04% (v v^−1^) Tween 20 alone (Supplementary Fig. 1; Negative control), so we consider it likely that this plant response is due to the initial mesophyll invasion by the mutant. While these results confirm an expected essential role of tubulin during plant colonisation, they illustrate the use of the P*nar1* promoter in studying essential genes during wheat infection.

### 3.4 P*gal7* and P*ex1A* are only partially repressed during colonisation but induced during pycnidiation

**Fig. 5:**
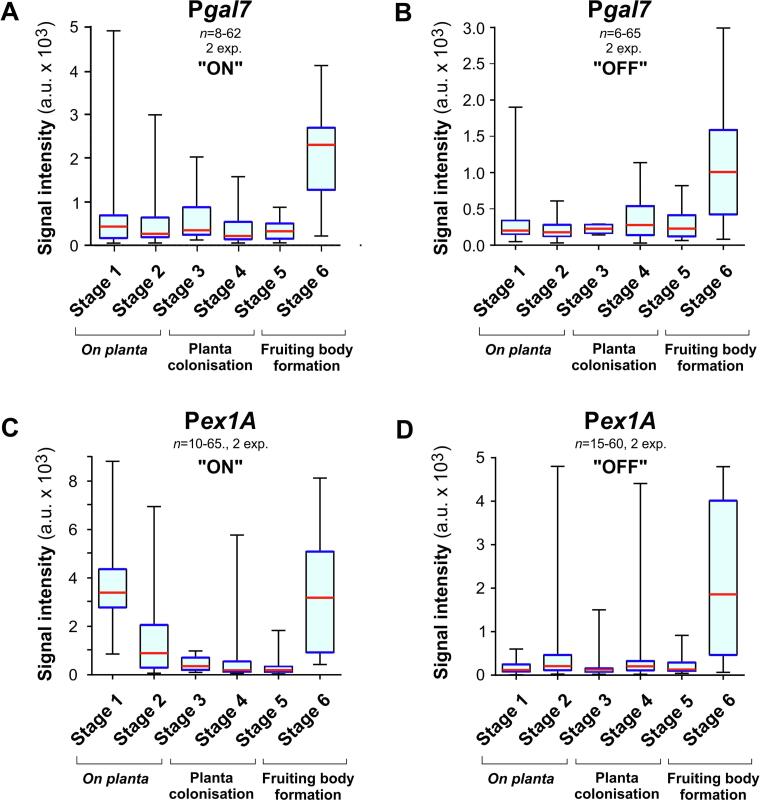
**The promoters P*gal7* and P*ex1A* are neither repressed, nor induced during the biotrophic phase of plant colonisation.** (**A**) Whiskers' plot showing average signal intensities of cytoplasmic eGFP, expressed under P*gal7*, normally controlling the expression of galactose-1-phosphate uridylyltransferase, in the six stages of wheat leaf infection. Note that plants were infected with cells that were grown in “ON” condition, where the inducing galactose was as sole carbon source. Sample size 8–62 structures from 2 experiments. (**B**) Whiskers' plot showing average signal intensities of cytoplasmic eGFP, expressed under P*gal7,* in the six stages of wheat leaf infection. Note that plants were infected with cells that were grown in “OFF” condition, with glucose being the sole carbon source. Even when grown under repression, the promoter shows significant activity, indicated by fluorescent signal from the reporter eGFP. This suggests that the fungus encounters plant cell wall-derived galactose during the colonisation of the plant. Sample size 6–65 structures from 2 experiments. (**C**) Whiskers' plot showing average signal intensities of cytoplasmic eGFP, expressed under P*ex1A* that normally controls 1,4-β-endoxylanase A, in the six stages of wheat leaf infection. Note that plants were infected with cells that were grown in “ON” condition, where expression-inducing xylose was provided as sole carbon source. Sample size 10–65 structures from 2 experiments. (**D**) Whiskers' plot showing average signal intensities of cytoplasmic eGFP, expressed under P*ex1A*, in the six stages of wheat leaf infection. Plants were infected with cells that were grown in “OFF” condition, with expression-repressing maltodextrin as sole carbon source. Even under this condition, the promoter does not tightly repress expression of the reporter eGFP, suggesting that the fungus encounters plant cell wall-derived xylose and/or arabinose during the colonisation of the plant. Sample size 15–60 structures from 2 experiments. As numerous data sets are non-normally distributed (Shapiro -Wilk test, P > 0.05), all data are given as Whiskers' plots (blue lines: 25/75 percentiles; red line: median, whiskers: minimum and maximum values). (For interpretation of the references to colour in this figure legend, the reader is referred to the web version of this article.)

The promoter P*gal7* is strongly repressed by glucose in liquid culture (Kilaru et al., 2015a; this study). This hexose sugar is found in the wheat leaf apoplast (Roman-Reyna & Rathjen, 2017) and is most likely produced by secreted PCWDEs during plant infection (Table 1). We tested the expression behaviour of P*gal7* during infection by growing strain IPO323_P*gal7*G_mChSso1 for 2 days in the presence of the inducer galactose (“ON” condition), followed by the infection of wheat leaves. Analysis of GFP signals revealed that P*gal7* was only partially repressed during plant infection, but induced in fruiting bodies (Fig. 5A; Stage 6). A similar result was obtained when *Z. tritici* spores were grown in the presence of glucose, which represses P*gal7* (Fig. 5B; “OFF” condition; Kilaru et al., 2015a). This suggests that fungal hyphae encounters galactose while colonising the mesophyll apoplast space. Galactose is a component of plant cell wall pectin and hemicellulases, such as arabinoxylan and arabinogalactans (overview in Scheller and Ulvskov, 2010). Galactose-producing fungal hemi-cellulases were reported to be expressed during infection (Table 1, Brunner et al., 2013, Rudd et al., 2015). At later stages of pathogenic development, when fruiting bodies are formed, *Z. tritici* switches to a necrotrophic phase, during which extensive plant cell death occurs (Kema et al., 1996, Keon et al., 2007, Kettles et al., 2017). At this stage, PCWDEs expression increases (Table 1) and increasing amounts of PCWDEs are found in the apoplastic fluid of *Z. tritici*-infected leaves (M'Barek et al., 2015). The increase in PCWDEs secretion during late infection likely releases more mono-saccharides, including galactose, from the plant cell wall. Thus, we consider it likely that the late induction of P*gal7* is due to the increased activity of secreted fungal hydrolases.

We tested if P*ex1A* is repressed *in planta* by growing *Z. tritici* cells of strain IPO323_P*ex1A*G_mChSso1 in the presence of the inducer xylose (“ON” condition) and infected 12 day-old wheat leaves. We found that the promoter was active on the plant surface (Stage 1, Fig. 5C) but only partially repressed during invasion of stomata and subsequent mesophyll colonisation (Stage 3–5; Fig. 5C). The inital activation was abolished in plants that were infected with *Z. tritici* cells, grown in “OFF” condition (Fig. 5D), suggesting that the early activation of P*ex1A* reflects the initial culture conditions rather than a specific activation. However, P*ex1A* was strongly induced during pycnidiation (Fig. 5C, 5D).

Our previous work has shown that P*ex1A* is induced by xylose (Kilaru et al. 2015a). Here, we show that arabinose can also activate this promoter. Both monosaccharides are part of arabinoxylans and other hemicelluloses in cereal plant cell walls (Burke et al., 1974, Hoffmann et al., 1992, Scheller and Ulvskov, 2010). *Z. tritici* expresses xylose-producing hemi-cellulases during all stages of wheat infection (Table 1), suggesting that the inducers xylose and arabinose are present during the biotrophic phase. We consider it likely that the slight induction of P*ex1A* in the biotrophic phase (Fig. 5C, 5D, Stage 3–5) is due to the competing inputs of inducing and repressing saccharides during apoplastic growth of the hyphae. The strong induction of P*ex1A* during fruiting body formation (Fig. 5C, 5D; Stage 6) is, again, most likely due to the increased secretion and activity of fungal PCWDEs during the necrotic phase of the infection cycle.

We need to add a word of caution here. While we conclude that increased activity of P*gal7* and P*ex1A* indicates elevated levels of galactose, arabinose or xylose, a study analysing the apoplastic metabolites in wheat leaves at late stages of infection does not agree with our findings (Keon et al. 2007). Instead, it revealed increased levels of glucose and fructose, which rather repress gene expression from P*gal7* and maybe P*ex1A*. Thus, we speculate that galactose, xylose or arabinose are produced locally to the hyphal surface. This could induce GFP gene expression without being recognised in nuclear magnetic resonance spectroscopy. Alternatively, regulation of P*gal7* and P*exA1 in planta* involves additional factors, which are not yet understood. Our results suggest that P*gal7* and P*ex1A* are not suitable to tightly repress genes during early stages of plant colonisation. However, the promoters could be used to induce over-expression of fungal genes during pycnidiation.

### 3.5 P*laraB* is strongly induced in planta

**Fig. 6:**
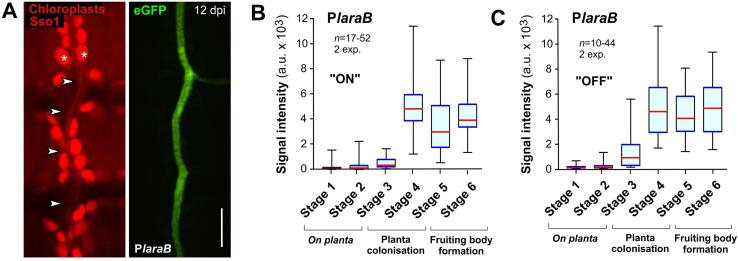
**The P*laraB* promoter is strongly induced during plant infection.** (**A**) Confocal images showing a hypha of strain IPO323_P*laraB*G_mChSso1 in wheat leaf tissue at 12dpi. The fungal plasma membrane is labelled with the red-fluorescent syntaxin mCherry-Sso1 (Kilaru et al., 2017; arrowheads in left image) and were co-detected with the auto-fluorescence of plant chloroplasts (asterisks in left panel). Due to induction of P*laraB*, cells express cytoplasmic eGFP (green). Scale bar represents 10 µm. (**B**) Whiskers' plot showing average signal intensities of cytoplasmic eGFP, expressed under P*laraB* that normally controls the expression of an α-l-arabinofuranosidase B, in the six stages of wheat leaf infection. Note that plants were infected with cells that were grown in “ON” condition, where arabinose was provided as a carbon source. Sample size 17–52 structures from 2 experiments. (**C**) Whiskers' plot showing average signal intensities of cytoplasmic eGFP, expressed under P*laraB*, in the six stages of wheat leaf infection. Note that plants were infected with cells that were grown in “OFF” condition, where glucose was provided as a carbon source. Sample size 10–44 structures from 2 experiments. As numerous data sets are non-normally distributed (Shapiro -Wilk test, P > 0.05), all data in (B, C) are given as Whiskers' plots (blue lines: 25/75 percentiles; red line: median, whiskers: minimum and maximum values). (For interpretation of the references to colour in this figure legend, the reader is referred to the web version of this article.)

In a final set of experiments, we tested the use of P*laraB* for repressing gene expression during plant infection. This promoter controls an α-l-arabinofuranosidase B and is induced by the presence of L-arabinose (“ON” condition), but largely repressed by glucose (“OFF” condition, Kilaru et al., 2015a; this study). To test if glucose in the apoplastic fluid represses P*laraB*, we grew cells of strain IPO323_P*laraB*G_mChSso1 in “ON” condition and infected 12 day-old wheat leaves. Surprisingly, analysis of GFP fluorescence in hyphae during the stages of infection revealed that P*laraB* is strongly induced during colonisation of the leaves mesophyll and in fruiting bodies (Fig. 6A, 6B, 6C; Stage 4–6). Thus, the fungus most likely encounters L-arabinose, which appears to “overwrite” the repressing effect of apolastic glucose. Arabinose is a structural part of arabinoxylans, which are a major component of the cell wall in cereal plants, including wheat (Burke et al., 1974, Dervilly-Pinel et al., 2001, Dervilly-Pinel et al., 2004). The *Z. tritici* genome encodes 4 α-I-arabinofuranosidases (JGI IDs: 70396, 68922, 111130, 40215). These enzymes hydrolyse plant cell wall polysaccharides, such as arabinoxylan or arabinogalactan, which releases L-arabinose. During infection of wheat, *Z. tritici* was shown to express α-I-arabinofuranosidases (Table 1), and 3 of these enzymes were also identified in the apoplastic fluid of colonised wheat leaves (M'Barek et al., 2015, Yang et al., 2015). Thus, we consider it most likely that the activity of secreted *Z. tritici* hydrolases produces the inducer of P*laraB in planta*.

## 4 Conclusion

In this study, we investigated 5 *Z. tritici* inducible/repressible promoters for their potential use in the evaluation of the role of essential proteins during wheat infection. Our primary goal was to test the suitability of conditional promoters for studying the cellular response of the pathogen to overexpression or repression of genes of interest. By introducing the promoter constructs into the defined *sdi1* locus (Kilaru et al., 2015b), we explicitly avoid the genomic context and associated regulatory aspects, which may differ between genes of interest. Moreover, our study focusses on individual cells within the plant tissue. In doing so, we also overcome another caveat of the infection process. Previous work has shown that wheat infection by *Z. tritici* is not synchronised (Fones et al., 2017). In fact, infected tissue can carry fungal cells in up to 4 different developmental stages at a given time (Fantozzi et al., 2020). Thus, quantification of gene expression from such leaves, using more “global” techniques (qRT-PCR, RNAseq), can only represent an average response of such mixed populations. In contrast, confocal microscopy allows investigation of responses of pathogen cells at specific developmental stages.

We reported previously that the promoters of nitrate reductase (P*nar1*), isocitrate lyase (P*icl1*), galactose-1-phosphate uridylyltransferase (P*gal7*), 1,4-β-endoxylanase A (P*ex1A*) and α-l-arabinofuranosidase B (P*laraB*) allow controlled gene expression by addition of nutrients or mono-saccharides in axenic culture. In particular P*ex1A* is tightly regulated in liquid cultures, which makes this promoter most suitable for the study of essential *Z. tritici* genes *in vitro*. However, the interaction between the pathogen and the host plant is of considerable significance for understanding infection strategies underpinning Septoria tritici blotch disease. Inside the plant, the invading hypha encounters plant-derived mono-saccharides, such as glucose (Roman-Reyna & Rathjen, 2017), but also produces potential inducing/repressing sugars due to the activity of secreted hydrolases (Table 1). To clarify which promoter may be suitable for molecular studies *in planta*, we therefore tested all 5 promoters for their usage in wheat. A quantitative analysis of GFP expression, driven from all 5 promoters, at 6 infection developmental stages revealed that only P*nar1* and P*icl*1 are repressed inside the plant (Stage 4, 5; Table 3). These promoters are therefore suitable to investigate the role of essential genes *in planta*. P*gal7* and P*ex1A* are only partially repressed, but strongly induced in pycnidia (Stage 6, Table 3), which may reflect the activity of apoplastic sugars, including those released from the plant cell wall by secreted fungal PCWDEs. Moreover, P*laraB* shows strong induction at all infection stages inside the plant (Stage 4, 5, 6; Table 3). Thus, P*gal7*, P*ex1A* and P*laraB* appear to be useful tools to investigate the effect of protein over-expression *in planta*. The power of such studies for understanding fungal plant infection was recently demonstrated in the smut fungus *Ustilago maydis*. Here, a combinatorial use of repressed and induced promoters revealed a role of early endosome motility in effector secretion during host-pathogen interaction (Bielska et al., 2014). Collectively, this work establishes conditional promoters for studying gene product function during host-pathogen interaction. In addition, it adds to the growing body of evidence that *Z. tritici* secretes PCWDEs during its biotrophic phase. This recently led to this phase being reclassified as the “latent necrotrophic” phase (Sanchez-Vallet et al., 2015).

**Table 3:** Transcriptional control by conditional promoters.

**Promoter**	***in vitro* expression**		***in planta* expression**	
	Induced	Repressed	Induced	Repressed
P*nar1*	Nitrogen	Ammonium	none	Stage 4, 5
P*icl1*	Sodium Aacetate	Glucose	Stage 1	Stage 4, 5
P*gal7*	Galactose	Glucose	Stage 6	none
P*ex1A*	Xylose/arabinose	Maltodextrin	Stage 6	none
P*laraB*	Arabinose	Glucose	Stage 4, 5, 6	none

Stage 1: Surface resting; Stage 2: Surface exploration; Stage 3: Stoma invasion; Stage 4: Mesophyll colonisation; Stage 5: Fruiting body initiation; Stage 6: Fruiting body maturation.

## CRediT authorship contribution statement

**Elena Fantozzi:** Investigation, Writing - original draft, Writing - review & editing. **Sreedhar Kilaru:** Resources. **Stuart Cannon:** Formal analysis, Data curation. **Martin Schuster:** Investigation. **Sarah J. Gurr:** Conceptualization, Writing - review & editing. **Gero Steinberg:** Conceptualization, Writing - original draft, Supervision, Project administration, Funding acquisition.

## Declaration of Competing Interest

The authors declare that they have no known competing financial interests or personal relationships that could have appeared to influence the work reported in this paper.
