# Supplementary material for: Conditional promoters to investigate gene function during wheat infection by Zymoseptoria tritici
Source: Fungal Genet Biol. 2021 Jan;146:103487. doi: 10.1016/j.fgb.2020.103487 (PMC7812376; doi:10.1016/j.fgb.2020.103487)
Supplement: Supplementary data 1 [file mmc1.docx]

**Supplementary Material**

**
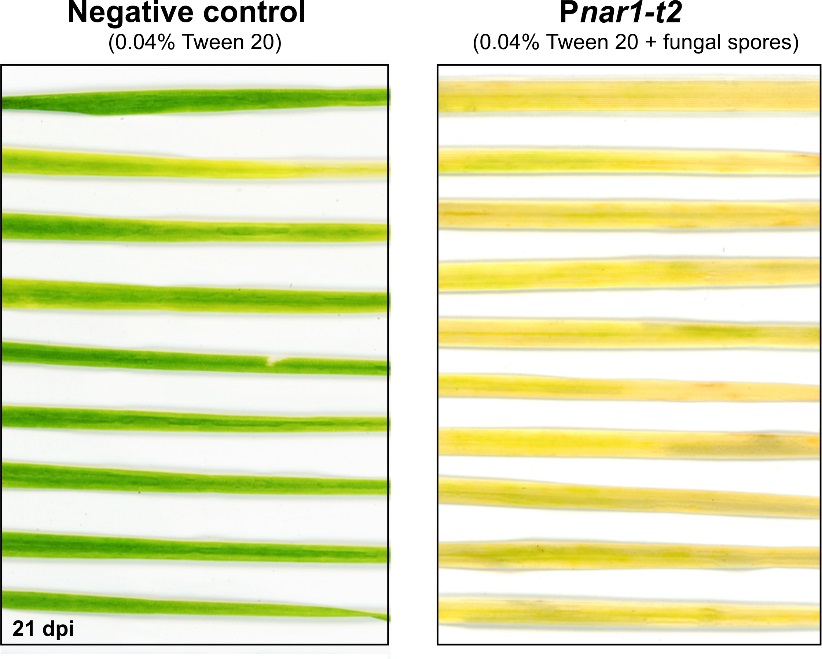
**

**Supplementary Figure 1.** Wheat leaves at 21 days after treatment with 0.04% (v v-1) Tween 20 (Negative control) and infection with strain IPO323_ZtG_P*nar1*Tub2 (P*nar1-t2*). Infection with the conditional α-tubulin mutant did not result in mature black pycnidia, but induced yellow chlorosis. Such discolouration is only rarely found in negative control leaves.

**Supplementary Table 1** Experimental usage of *Z. tritici* strains

| **Strain name Figure** | | |
| --- | --- | --- |
| IPO323_P*nar1*G_mChSso1 | Figures: 3A, 3B, 3C, 3F |  |
| IPO323_P*icl1*G_mChSso1 | Figures: 2A, 3D, 3E, 3F |  |
| IPO323_G | Figure: 3F |  |
| IPO323_ZtG | Figures: 4B, 4C, 4D, 4E, 4F |  |
| IPO323_ZtG_P*nar1*Tub2 | Figures: 4B, 4C, 4D, 4E, 4F; Suppl. Fig. 1 |  |
| IPO323_P*gal7*G_mChSso1 | Figures: 2A, 5A, 5B |  |
| IPO323_P*ex1A*G_mChSso1 | Figures: 2A, 2B, 5C, 5D |  |
| IPO323_P*laraB*G_mChSso1 | Figures: 2A, 6A, 6B, 6C |  |

**Supplementary Table 2** Primers used in this study

| **Primer name Sequence (5’ to 3’)** | |
| --- | --- |
| SK-Sep-136 | CCCAACTGATATTGAAGGAGCATT |
| SK-Sep-137 | CCCGATCTAGTAACATAGATGACA |
| SK-Sep-217 | *TGGCAGGATATATTGTGGTGTAAACAAATT*GCAGTCGACGCCAGATGATGG |
| SK-Sep-218 | CCAAAAAATGCTCCTTCAATATCAGTTGGG*GGCGATGGTGGTATGCGGATG* |
| SK-Sep-219 | *GCGCGGTGTCATCTATGTTACTAGATCGGG*ATAGTTGCTCTACGACCAATGCC |
| SK-Sep-220 | *CGTACCGTTCAAGGAGATGACTTCACGCAT*TGCGGGAGAGGACATAGTAACG |
| SK-Sep-221 | ATGCGTGAAGTCATCTCCTTGAAC |
| SK-Sep-222 | *TAAACGCTCTTTTCTCTTAGGTTTACCCGC*TGGGACGCTCGATGCCAAGGTT |
| Italics indicate sequence complementary with another DNA fragment, which allows homologous recombination in *S. cerevisiae*. | |
